# Genome-wide association study of antipsychotic-induced sinus bradycardia in Chinese schizophrenia patients

**DOI:** 10.1371/journal.pone.0249997

**Published:** 2021-04-29

**Authors:** Saizheng Weng, Bo Wang, Mo Li, Shan Chao, Ruiqian Lin, Rongyan Zheng, Yinliang Yu, Shaonan Guo, Xianhao Lin

**Affiliations:** 1 Fuzhou Neuro-Psychiatric Hospital Affiliated to Fujian Medical University, Fuzhou, Fujian Province, China; 2 Shenzhen Baoan Women’s and Children’s Hospital, Jinan University, Shenzhen, Guangdong Province, China; 3 Bio-X Institutes, Shanghai Jiaotong University, Shanghai, China; 4 Institutes for Shanghai Pudong Decoding Life, Shanghai, China; 5 School of Health, Fujian Medical University, Fuzhou, Fujian Province, China; University of North Carolina at Chapel Hill, UNITED STATES

## Abstract

Second-generation antipsychotics (SGAs) play a critical role in current treatment of schizophrenia (SCZ). It has been observed that sinus bradycardia, rare but in certain situations life threatening adverse drug reaction, can be induced by SGAs across different schizophrenia populations. However, the roles of genetic factors in this phenomenon have not been studied yet. In the present study, a genome-wide association study of single nucleotide polymorphisms (SNPs) was performed on Chinese Han SCZ patients to identify susceptibility loci that were associated with sinus bradycardia induced by SGAs. This study applied microarray to obtain genotype profiles of 88 Han Chinese SCZ patients. Our results found that there were no SNPs had genome-wide significant association with sinus bradycardia induced by SGAs. The top GWAS hit located in gene *KIAA0247*, which mainly regulated by the tumor suppressor P53 and thus plays a role in carcinogenesis based on resent research and it should not be a susceptibility locus to sinus bradycardia induced by SGAs. Using gene-set functional analysis, we tested that if top 500 SNPs mapped genes were relevant to sinus bradycardia. The result of gene prioritization analysis showed *CTNNA3* was strongly correlated with sinus bradycardia, hinting it was a susceptibility gene of this ADR. Our study provides a preliminary study of genetic variants associated with sinus bradycardia induced by SGAs in Han Chinese SCZ patients. The discovery of a possible susceptibility gene shed light on further study of this adverse drug reaction in Han Chinese SCZ patients.

## Introduction

Schizophrenia (SCZ) is a complex and highly heritable psychiatric disorder with a relatively high prevalence of nearly 7.2/1,000 for lifetime morbid risk [1, 2]. SCZ is characterized by emotional dysregulation, positive and negative symptoms, psychosis and cognitive impairment [3, 4]. Several treatments for SCZ exist, but SCZ is difficult to cure completely and high recurrence and disability rates have been noted among SCZ patients. In particular, this disease has a considerable adverse influence on work, life and study of patients. Although previous studies have shown that multiple factors are involved in the pathogenesis of SCZ, including hereditary factors, environmental factors and other neurological changes in brain [5, 6], the exact pathophysiological mechanisms of SCZ still remain unclear.

Drug therapy is currently the most effective treatment for SCZ patients. Second-generation antipsychotics (SGAs) are widely used in clinical treatment because their numerous advantages, including wider application spectrum, greater potency, fewer extrapyramidal side effect and proved improvement of the quality of life of SCZ patients [7]. However, adverse drug reactions (ADRs), including weight gain, metabolic syndrome, tardive dyskinesia (TD), elevated prolactin level and others [8–10], will appear in SCZ patients after taking SGAs. ADRs vary from person to person, and this variation may be attributed to the genetic variation between individuals. In the post-genome era, it is important to explore the mechanisms that drive these differences and exploit them to predict drug response using the individual-specific genetic background. TD is the most thoroughly studied antipsychotic-induced ADR, and many single nucleotide polymorphisms (SNPs) have been associated with it, such as Ser311Cys in *DRD2*, Ser9Gly in *DRD3*, T102C and -1438G/A in *HTR2A* [11]. Recently, the goal of drug therapy has shifted from emphasizing solely on controlling psychotic symptoms and impulsive behavior to focusing on social functional recovery and on reducing ADRs [12].

In previous studies, it has been observed that subpopulations of SCZ patients manifested a significant decrease in resting heart rate and disease progressed to sinus bradycardia after taking SGAs, while other patients taking the same medicines did not show any abnormalities in heart rhythm. Similar phenomena have been reported by researchers around the world without studying the mechanisms that drive them [13]. Sinus bradycardia is defined as a mean sinus rate of less than 60 beats per minute and is a commonly observed arrhythmia. It could be either physiologic, as a normal phenomenon in young athletic individuals or during sleep, or pathologic, driven intrinsic cardiac and other pathological conditions [14]. Usually, the occurrence of sinus bradycardia does not require intervention given the overlap of heart rate ranges with non-pathologic changes, and it is being assessed case by case. However, persistent severe sinus bradycardia can lead to sudden cardiac death. Several studies have recently discovered causal genes for diseases with clinical symptoms of sinus bradycardia through pedigree analysis, linkage analysis or association studies. Most of these genes are cardiac ion channel genes (e.g. *HCN4*, *SCN5A*, *RYR2*) or core genes participating in the regulation of heart morphogenesis, such as *MYH6* or *SHOX2* [15–19]. To the best of our knowledge, there are no studies exploring the roles of genetic factors in sinus bradycardia caused by SGAs. In view of the prevalence of sinus bradycardia in SCZ patients after taking SGAs and the potential harm of patients’ health, it is necessary to perform studies to explore common susceptibility gene or loci associated with abnormal heart rate or sinus bradycardia induced by SGAs. These data could allow personalizing the treatment of patients.

In the present paper, we present a preliminary case-control genome-wide association study (GWAS) to identify genomic variants associated with sinus bradycardia induced by SGAs. We aimed to 1) detect whether common variants play a role in the development of ADR during SGA treatment; 2) identify potential candidate genes for ADR in SCZ patients. Finally, we identified possible meaningful variants and genes associated with this ADR, and this finding is an elementary contribution for promoting our understanding on this phenomenon.

## Materials and methods

### Patient samples

Patients were recruited from Fuzhou City in Fujian Province, China from September 2018 to September 2019 and all belonged to Chinese Han nationality. SCZ of the patients was diagnosed according to the criteria of the Diagnostic and Statistical Manual of Mental Disorders, Fifth Edition (DSM-V). All subjects met the following criteria: 1) absence of any abnormal heart rhythm before taking SGAs; 2) no history of congenital heart disease; 3) not any other organic changes of heart. A total of 90 patients were eventually recruited to our study. All participants were being administered with only one kind of SGAs, and 33 patients developed sinus bradycardia with resting heart rate < 60 beats per minute after taking SGAs. The latter consisted of the sinus bradycardia group of our study. The remaining 57 patients with no clinical manifestations of sinus bradycardia after taking SGAs made up the control group.

This study was approved by the Ethical Committee of Fuzhou Neuro-psychiatric Hospital affiliated to Fujian Medical University (2018-LS-NO.6). All subjects or their legal guardians understood the procedures and provided written informed consent prior to their enrollment in this study according to the Declaration of Helsinki [20]. The authors had access to information that could identify individual participants during or after data collection.

### DNA genotyping, quality control and imputation

Peripheral whole blood samples were collected in EDTA-vacutainer tubes and stored at -80°C until DNA extraction. Genomic DNA was extracted using a QIAamp DNA Blood Kit (Qiagen) and then stored at -20°C. DNA concentration and purity was measured with a NanoDrop 2000c UV-Vis spectrophotometer (Thermo Scientific), and DNA integrity was identified by electrophoresis using 0.8% agarose gel.

DNA samples were genotyped using the Illumina Infinium Global Screening Array-24 v1.0 with 200 ng of genomic DNA, and original data were collected using Illumina’s GenomeStudio v2.0 software according to the manufacturer’s instructions. Then genotypes were aligned to the Human Genome built version 37, and exported to PLINK format data for the next quality control using PLINK V1.9 [21].

**Fig 1:**
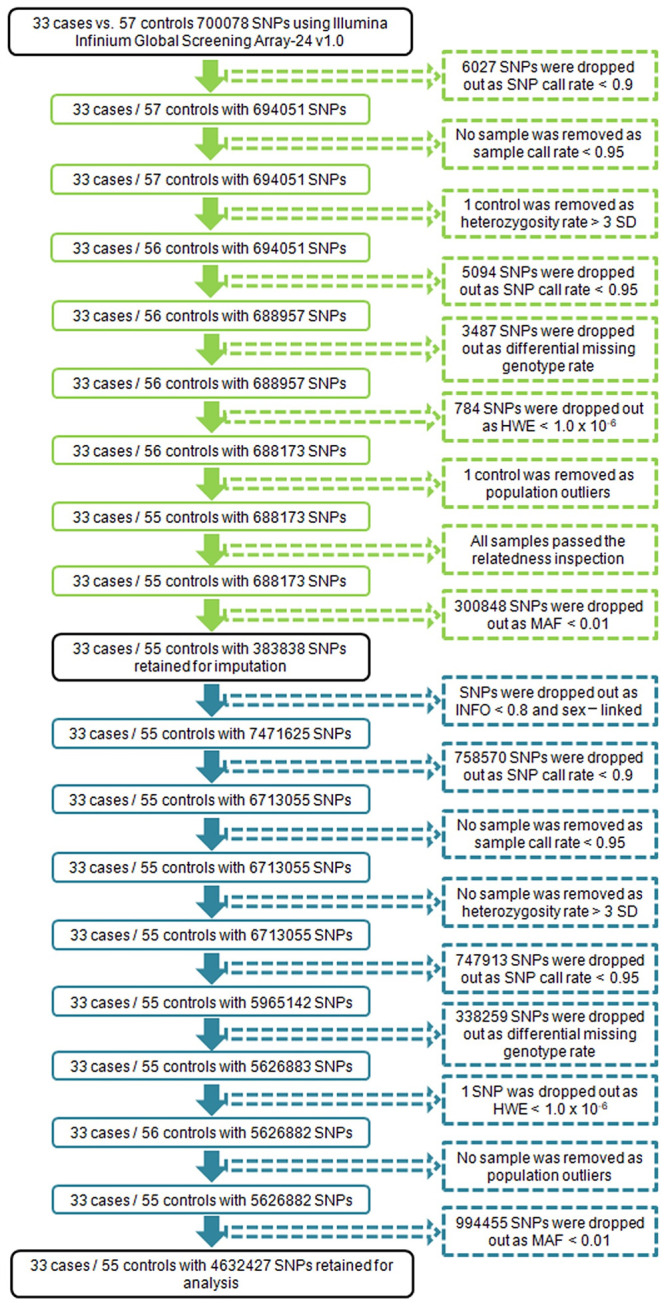
Workflow of the quality control process of genotypes data. The pre-imputation quality control steps are marked in green, and 383,838 SNPs passed the criteria. The post-imputation quality control processes are marked in blue.

Pre-imputation quality control was performed as following steps: (1) excluding SNPs with a call rate < 90%; (2) excluding subjects with a sample call rate < 95%; (3) excluding subjects with heterozygosity rate > 3 standard deviations of sample mean; (4) excluding SNPs with a call rate < 95%; (5) excluding SNPs with a differential missing genotype rate > 5% or < -5% between cases and controls; (6) excluding SNPs with a Hardy Weinberg Equilibrium p value < 10^−6^; (7) the samples passed above filtering criteria were used to principal component analysis (PCA), further one sample was removed as population outlier, because its eigenvector value > 3 standard deviations of sample mean on the second principal components axis; (8) excluding samples with a mismatch in the computationally inferred sex and the reported clinical sex; (9) excluding SNPs with a minor-allele frequency < 1%. In addition to the above quality control steps, we checked relatedness of the left samples through calculating identity-by-descent (IBD) with a cutoff < 0.1875, but no samples failed to pass the criterion. The numbers of SNPs and samples removed in each step are shown in Fig 1.

Genotype imputation was performed to capture missing genotypes using all haplotypes from the 1000 Genomes Project Phase 3 version 5 reference panel [22]. Pre-phasing was carried out in SHAPEIT2 [23], strand alignment and reference alleles harmonization were resolved using Genotype harmonizer [24]. Imputation was performed with IMPUTE2 [25]. First, imputed SNPs with INFO score < 0.8 and sex-linked were excluded. The following post imputation filter steps and parameters were same with pre-imputation quality control except steps checking sex mismatch and relatedness. Detailed processes are also shown in Fig 1. The final dataset included genotypes for 4,632,427 SNPs.

### Functional analysis

Top 500 SNPs were annotated with different databases and listed in S1 Table, and the mapped genes were listed in S2 Table and selected for following functional analysis, including gene prioritization analysis, gene ontology (GO) enrichment analysis and protein-protein interaction (PPI) network analysis.

Gene prioritization analysis was performed using the online tool ToppGene in the ToppGene Suite (https://toppgene.cchmc.org/), which is a portal that allows gene list enrichment analysis and candidate genes prioritization [26]. The tool ToppGene prioritizes candidate genes based on functional similarity of at most 19 features to training gene list, which consists of genes known to be related to the disease of interest. Similarity scores from individual features are combined into an overall score using statistical meta-analysis and are real values in [0,1], however for a missing value, the score is set to -1 [26]. First, to generate the “training gene list” of genes for sinus bradycardia, we conducted a search in database Online Mendelian Inheritance in Man (OMIM) and human phenotype ontology (HPO) using keyword of “sinus bradycardia”, and 16 genes and 21 genes were retrieved from these two databases, respectively. After review by some authors and elimination of duplicate genes, the final gene list consisted of 29 genes, including *KCNE1*, *HCN4*, *SCN5A*, *GNB5*, *MYH6*, *KCNH2*, *KCNJ2*, *ANK2*, *CACNA1D*, *MYL4*, *SCN4B*, *PRKAG2*, *KCNQ1*, *PPA2*, *RYR2*, *SHOX2*, *AKAP9*, *BVES*, *CACNA1C*, *CALM1*, *CALM2*, *CALM3*, *CAV3*, *KCNE2*, *KCNJ5*, *SCN10A*, *SNTA1*, *TNNI3K* and *TRDN* genes. Next, this “training gene list” was used in ToppGene simultaneously with the “test gene list”—top 500 SNPs mapped genes except 5 genes, *RP11-362A1*.*2*, *snoU13*, *CTD-2066L21*.*3*, *CTB-35F21*.*1* and *RP11-402L1*.*11*, which could not match any approved gene symbol of HGCN Symbol database in step of “input normalization”, and prioritization analysis based on annotation was performed through a fuzzy-based multivariate approach with the following features: Gene Ontology terms for molecular function, biological process and cellular component, human and mouse phenotype, pathway, pubmed, coexpression, coexpression atlas and disease. The method used to determine statistical significance (*P* value < 0.05) of training genes was the Hypergeometric Probability Mass Function with Benjamini-Hochberg False_Discovery Rate (FDR) correction. The overall *P* value of every test gene was obtained as previous description [27].

GO enrichment analysis provides a framework to annotate and classify genes based on three categories, including biology process (BP), molecular function (MF), and cellular component (CC) [28]. The module Functional Annotation Chart of Database for Annotation, Visualization, and Integrated Discovery (DAVID) 6.8 (https://david.ncifcrf.gov/) was used for GO enrichment analysis with a statistically significant cutoff of *P* value < 0.05. The top 10 terms of GO analysis results were visualized by package “ggplot2” of *R* software.

The Search Tool for the Retrieval of Interacting Genes/Proteins (STRING; https://string-db.org/) is a database of known and predicted protein-protein interactions including direct (physical) and indirect (functional) associations. The latest version of STRING v11.0 more than doubles the number of organisms against previous version, to 5090 [29]. The PPI analysis was performed and visualized using STRING v11.0. Only the protein-coding genes were included the PPI analysis, and these genes were listed in S3 Table. The statistical background for PPI analysis was assumed at whole genome level, and FDR < 0.05 was considered statistically significant.

### Statistical analysis

The genetic associations between the genome-wide variants and sinus bradycardia induced by SGAs were tested by deploying logistic regression analysis under an additive model with adjustment for the first five principal components which were revealed from PCA with the–pca function of PLINK. The GWAS analysis was conducted using PLINK V1.9. A *P* value significance threshold of 5.0 × 10^−8^ was used to call genome-wide significant associations, while SNPs with a *P* value ranging between 1.0 × 10^−5^ and 5.0 × 10^−8^ were considered as variants showing a suggestive association. A Manhattan plot of -log_10_ (*P* value) was generated using package “qqman” of *R* software. Significant differences of demographic characteristics between sinus bradycardia group and control group were analyzed using student’s *t*-test for data of age and heart rate which could be statistically analyzed using parametric tests and Chi-square test for other characteristics which could be statistically analyzed using non-parametric tests in SPSS software (version 11.0, Chicago, IL, USA), to confirm the homogeneity of the data used in our analyses. These results are presented in Table 1. *P* < 0.05 was considered as the threshold for inferring statistical significance.

**Table 1:** Demographic characteristics of the study patients.

Characteristics		All patients (N = 88)	Adverse drug response		
			Non-sinus bradycardia (N = 55)	Sinus bradycardia (N = 33)	*P* value
Age	Mean ± SD	41.7 ± 14.0	45.1 ± 14.2	36.1 ± 12.0	0.003^a^
Sex	Female	36 (40.9%)	26 (47.3%)	10 (30.3%)	0.179^b^
	Male	52 (59.1%)	29 (52.7%)	23 (69.7%)	0.179^b^
Heart rate	Mean ± SD	66.9 ± 15.2	76.4 ± 11.0	51.2 ± 3.9	2.20E-16^a^
Antipsychotics	Risperidone	16 (18.2%)	11 (20.0%)	5 (15.2%)	0.775^b^
	Olanzapine	39 (44.3%)	29 (52.7%)	10 (30.3%)	0.202^b^
	Amisulpride	25 (28.4%)	9 (16.4%)	16 (48.5%)	0.003^b^
	Ziprasidone	2 (2.3%)	1 (1.8%)	1 (3.0%)	1^b^
	Aripiprazole	4 (4.5%)	3 (5.5%)	1 (3.0%)	1^b^
	Quetiapine	2 (2.3%)	2 (3.6%)	-	0.712^b^

SD, standard deviation.

^a^ Student’s t-test,

^b^ Chi-square test.

## Results

### Characteristics of samples

The demographic characteristics of the participants of the study are summarized in Table 1. The mean age of all 88 patients, 36 women and 52 men, was 41.7 years old. Thirty-three of the 88 patients developed sinus bradycardia after taking SGAs, with an average resting heart rate and age of 51.2 beats per minute and 36.1 years old, respectively. Significant differences between sinus bradycardia group and control group were observed in resting heart rate and age. The SGAs of patients were groups as follows: 16 patients taking risperidone, 39 olanzapine, 25 amisulpride, 2 ziprasidone, 4 aripiprazole and 2 quetiapine. Furthermore, statistically significant differences were also observed in the number of patients taking amisulpride.

### Genome-wide association study (GWAS) of genotyped and imputed SNPs using 1000 Genomes Project data

A case-control GWAS analysis was performed to identify SNPs associated with sinus bradycardia induced by SGAs. We initially obtained 700,078 SNPs of peripheral blood cells using microarray measurements in 90 schizophrenia (SCZ) patients. After the pre-imputation quality control steps shown in Fig 1 and marked in green, 383,838 SNPs from 88 schizophrenic patients were selected for the follow-up analyses. Genotype imputation was applied for filtering SNPs data using 1000 Genomes Project data Phase 3 version 5 reference panel, and another quality control process shown in Fig 1 and marked in blue was subsequently applied to alleviate the problem of missing genotypes data. Finally, we acquired 4,632,427 SNPs excluding SNPs from sex chromosomes to perform GWAS analysis.

**Fig 2:**
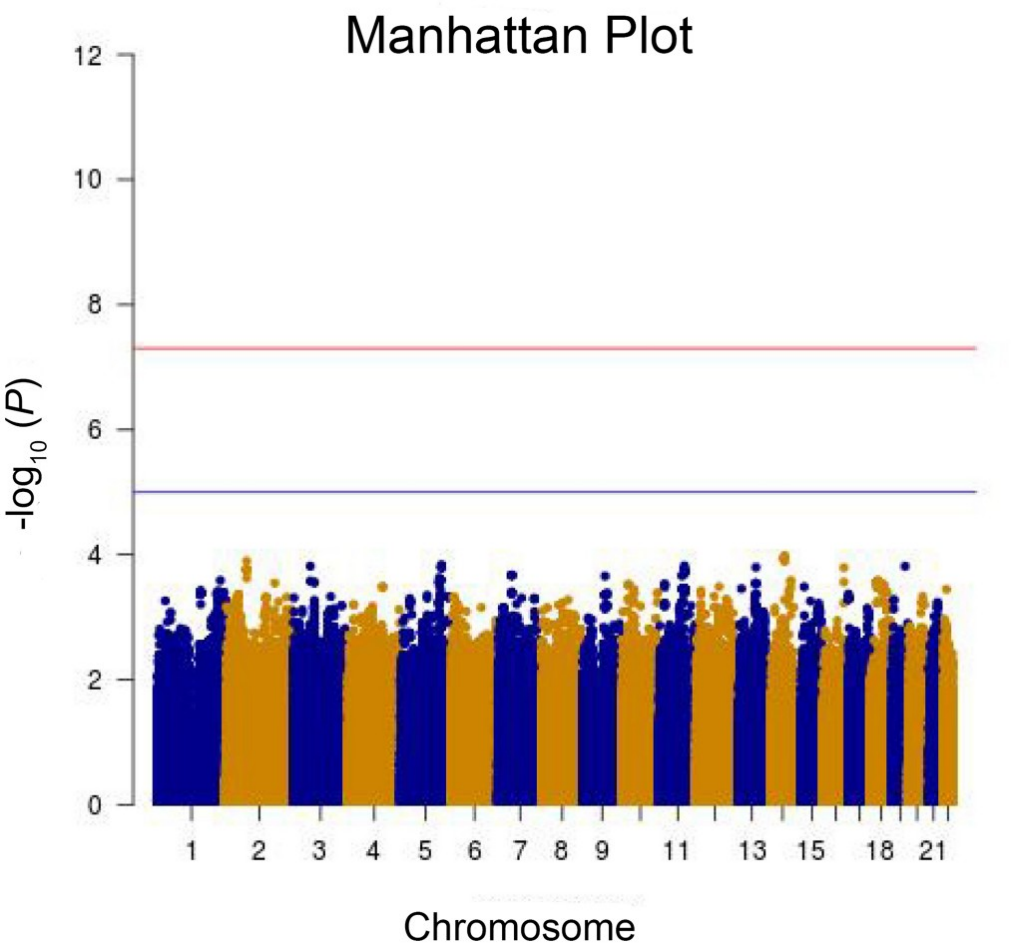
Manhattan plot of Genome-wide association study. Manhattan plot for genetic association study of sinus bradycardia induced by SGAs. Blue and orange dots represent SNPs within odd and even chromosomes, respectively. Red and blue line represents genome-wide-significance threshold (*P* < 5.0× 10^−8^) and suggestive significance threshold (*P* < 1.0 × 10^−5^), respectively.

GWAS analysis based on logistic regression adjusting for the first five eigenvectors from PCA was performed on the 4,632,427 SNPs to identify the risk genetic variants explaining individual differences in effects on heart rate of SGAs. Manhattan plot is depicted in Fig 2, and none of the examined SNPs were found to be significant at the genome-wide level (*P* < 5 × 10^−8^) or even at the suggestive level (*P* < 1 × 10^−5^). The top 500 SNPs from GWAS analysis were listed in S1 Table and annotated with different databases. The top GWAS hit was SNP rs1275749 located in the intron of gene *KIAA0247* with a *P* value of 0.0001032, meanwhile there were other loci within the KIAA0247 gene ranked high in the GWAS result (S1 Table). However, *KIAA0247* mainly plays a role in carcinogenesis [30], and there is no evidence to support the role of *KIAA0247* in the development of sinus bradycardia.

### Gene set enrichment and prioritization analyses

In view of the absence of any significant risk SNPs associated with sinus bradycardia induced by SGAs, we attempted to identify if there were genes associated with sinus bradycardia through gene set functional analyses. The genes annotated from top 500 independent SNPs ranked by *P* value were selected, and a gene set consisting of 60 genes encoding protein or non-coding RNA (ncRNA) was revealed and summarized in S2 Table.

**Fig 3:**
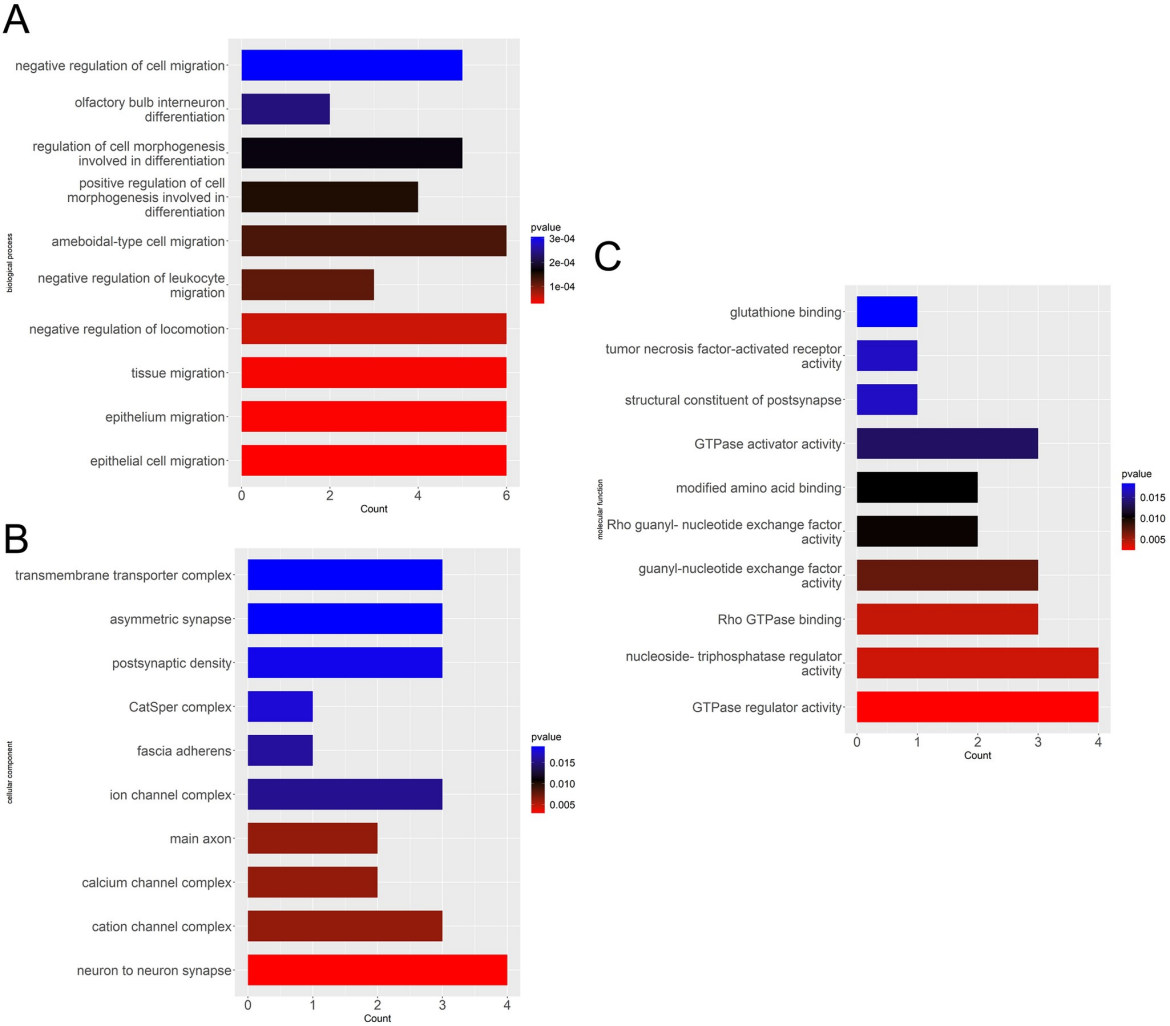
GO enrichment analyses of top 500 SNPs mapped genes. Top 500 SNPs mapped genes are classified into three functional categories, including biological process (A), cellular component (B) and molecular function (C). The ordinate axis represents different functional terms, and the abscissa axis represents the number of enriched genes. Different colors indicate different *P* values, Red: smaller *P* value, Blue: bigger *P* value.

To analyze the molecular and functional characteristics of top 500 SNPs mapped genes, gene ontology (GO) enrichment analysis was subsequently performed only in 38 genes encoding protein, and results of GO analysis under three categories of biological process (BP), molecular function (MF) and cellular component (CC) with the *P* value < 0.05 were summarized in S4 Table, and top 10 terms of the three categories were visualized in Fig 3, respectively. As shown in Fig 3, these genes were particularly enriched in migration and its regulation of cell or tissue and regulation of cell morphogenesis during differentiation within BP category (Fig 3A), while enriched in biological compound binding activity and enzyme regulator activity under MF category (Fig 3C). The most interesting finding was obtained from the CC category, the 38 genes were significantly enriched in component of ion channel or transporter complex, which was highlighted in pathogenesis of sinus bradycardia, and axon or synapse.

Next, we retrieved genes associated with the clinical manifestation of sinus bradycardia in the OMIM and HPO databases, inputted them as training gene list into ToppGene tool in ToppGene Suite, and prioritized the top 500 SNPs mapped genes in order to further analyze their potential association with sinus bradycardia by calculating similarity scores between the training genes and our top 500 SNPs mapped genes. The training genes showed a strong correlation with sinus bradycardia based on functional analysis results of different features, such as GO term, human phenotype and disease (S5 Table). Therefore, it was reasonable to prioritize top 500 SNPs mapped genes with this computational model. Finally, a total of 55 genes were included in gene prioritization analysis, and the results of gene prioritization were listed in S5 Table. Table 2 presents the list of top 19 prioritized genes related to sinus bradycardia with an overall *P* value less than 0.05. Two genes *RYR3* and *CTNNA3* showed the highest correlation priority to sinus bradycardia, due to average scores more than 0.8 and overall *P* values less than 1× 10^−5^. Average scores of other 17 genes ranged between 0.2 and 0.7 (Table 2).

**Table 2:** Priority list of potential association with sinus bradycardia of top 500 SNPs mapped genes.

Rank	Gene Symbol	Average Score	Overall *P* Value
1	*RYR3*	0.842	1.25E-06
2	*CTNNA3*	0.807	6.93E-06
3	*DLG2*	0.518	0.0003519
4	*SYT1*	0.647	0.0004599
5	*AKT3*	0.578	0.0007583
6	*ESRRG*	0.535	0.001094
7	*NEDD4*	0.538	0.001432
8	*SLIT2*	0.508	0.001902
9	*CNTN4*	0.462	0.01026
10	*ROBO2*	0.389	0.01302
11	*ABR*	0.504	0.01544
12	*PTPRG*	0.377	0.0172
13	*PSD3*	0.294	0.02446
14	*GLRX*	0.426	0.02584
15	*TNFRSF11A*	0.36	0.02594
16	*ZEB2*	0.423	0.02676
17	*MGST1*	0.424	0.03444
18	*FA2H*	0.338	0.03454
19	*DOCK1*	0.444	0.0423

**Average score**: an overall score combining similarity scores from individual feature using statistical meta-analysis. **Overall *P* value**: statistical significance values of average score.

### Protein-protein interaction network analysis

**Fig 4:**
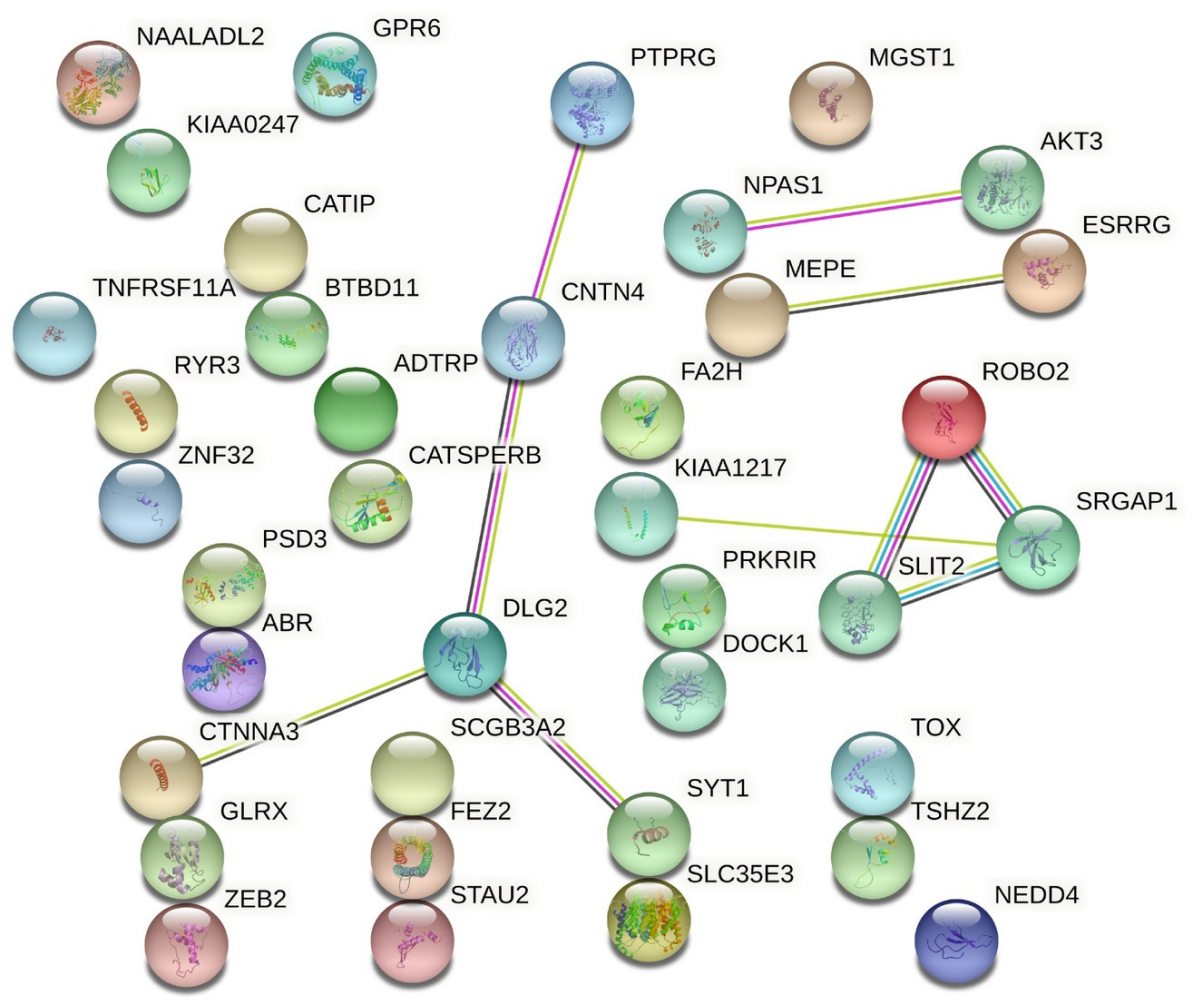
Protein-protein interaction (PPI) network analysis. PPI analysis was performed and visualized using the online tool STRING. Proteins are represented as nodes. Different colored lines represent types of evidence used for predicting PPIs (light blue line: known interaction from curated databases; pink line: experimentally determined evidence; grass green line: textmining evidence; black line: co-expression evidence).

A growing number of studies have shown that protein-protein interactions (PPI) play an important role in the development of diseases [31, 32], and the analysis of PPI can suggest risk genes/proteins of diseases. Next, we analyzed the PPI with 38 protein-coding genes listed in S3 Table using online tool STRING v11.0. To construct the PPI network in STRING, the accession numbers of the 38 genes were copied into the search box using the Multiple Proteins module, and Homo sapiens was chosen as the organism. As shown in Fig 4, the interactions between these genes were minimal, and there were also no significant hub gene. Still, interactions among three genes *ROBO2*, *SLIT2* and *SRGAP1* were reliable and supported by evidences from databases and experiment. And *DLG2* could directly interact with two genes *CNTN4* and *SYT1*, confirmed by experiments. Interestingly, *CTNNA3*, having a strong correlation with sinus bradycardia backed up by our previous analysis, also interacted with *DLG2* through circumstantial evidences. Other interactions were observed between *NPAS1* and *AKT3*, *MEPE* and *ESRRG*.

## Discussion

The second-generation antipsychotics (SGAs) have adverse drug reactions (ADRs) on the central nervous system, metabolic system, endocrine system and cardiovascular system [33, 34], and the occurrence of these ADRs is partly owed to hereditary factor. Sinus bradycardia is a rare ADR induced by SGAs, and a thorough understanding of the underlying hereditary factor is not available for this phenomenon.

Our study was the first attempt to interpret the hereditary factors of this ADR by GWAS analysis. The SNP profiles of 33 SCZ patients with sinus bradycardia and 55 unaffected SCZ patients were genotyped with microarray, and the genetic associations between SNPs and sinus bradycardia were measured by deploying logistic regression analysis. The top hit of GWAS analysis was a SNP rs1275749 with an odd ratio of 9.622 (3.069–30.17, 95% confidence interval) located in the intron region of the *KIAA0247* gene (S1 Table), meanwhile in the *KIAA0247* gene range, there were other SNPs with minimal *P* value. The rs1275749 was only studied in GWAS of cancers, neurodegenerative diseases and metabolic disease in GWAS Central database, in addition *KIAA0247* is a novel and highly conserved gene, which plays a critical role in oncogenesis because of a p53-responsive element found in its promoter region [30]. Nevertheless, recent research results highlight the importance of ion channel dysfunction during the development of sinus bradycardia [35, 36]. Thus, it is easily and rationally inferred that there is no correlation between these SNPs and sinus bradycardia induced by SGAs based on current knowledge, and *KIAA0247* is also not a susceptibility gene for this ADR. This may be not a true result caused by our small sample size.

In addition we did not find any other statistically significant gene loci associations with sinus bradycardia below genome-wide significance (*P* < 5 × 10^−8^) or even suggestive significance (*P* < 1 × 10^−5^), mainly due to the small sample size and using Bonferroni corrections in a such small sample set, a strict method for multiple testing corrections in this GWAS analysis. Another reason may be that SNPs obtained from gene microarray were common genetic variations and the contribution of common variations to this ADR is characterized as moderate.

For further analysis and interpretation of the GWAS results, top 500 SNPs mapped genes were subjected to do functional analysis, including GO enrichment analysis, gene prioritization analysis and PPI analysis.

In GO enrichment analysis, three genes, namely *DLG2*, *CATSPERB* and *RYR3* (S4 Table), were enriched in ion channel complex under cellular component. From a purely functional point of view, this function is very similar to the pathogenesis of sinus bradycardia. However, *DLG2* was also grouped into “main axon” term and function of this gene product is to mediate signal transduction by forming a heterodimer at postsynaptic sites, not supporting this gene as a susceptibility gene for this ADR. *CATSPERB* is probably involved in sperm cell hyperactivation and also not regarded as a susceptibility gene. *RYR3* encodes a ryanodine receptor and it is involved in releasing calcium from intracellular storage following stimulation. However, *RYR3* is mainly expressed in skeletal muscle and brain [37], while one of its important paralogs is *RYR2*, which is primarily being expressed in the heart and brain [38]. Mutations in *RYR2* could induce bradycardia in patients with catecholaminergic polymorphic ventricular tachycardia (CPTV) [39]. Therefore, all three genes *DLG2*, *CATSPERB* and *RYR3* may not serve as susceptibility genes of sinus bradycardia induced by SGAs. Results from gene prioritization analysis highlight two genes*RYR3* and *CTNNA3* that may play a role in sinus bradycardia induced by SGAs. *RYR3* has been excluded from the susceptible gene by the above analysis. While *CTNNA3* encodes a protein that belongs to the vinculin/alpha-catenin family, and highly expressed in heart tissues [40]. To date, the exact function of CTNNA3 is still not clear, but previous studies have shown that CTNNA3 could bind with plakophilins to form a combination of desmosomal and adherens junctional proteins involved in cell-cell adhesion of cardiomyocytes [41], and mutations in this gene, such as a missense mutation Val94Asp and an in-frame 3-bp deletion, are associated with arrhythmogenic right ventricular dysplasia, familial 13 (ARVD13) [41]. But only one SNP rs10762127 is located in *CTNNA3* from our top 500 SNPs, and the odd ratio and *P* value of rs10762127 are 4.065 (1.813–9.114, 95%CI) and 0.000664 (S1 Table), respectively. Our analysis results suggested that we could not find evidence of significant association between rs10762127 and this ADR in our small sample, and this association should be retested and validated in another large or multi stages case-control study. Finally, the PPI analysis does not provide more useful information about our results, except for the interaction between *CTNNA3* and *DLG2* supported by circumstantial evidences. As mentioned above, *DLG2* is mainly expressed and functions in synapse, while *CTNNA3* is primarily expressed in heart, thus we inferred that *DLG2* was not a susceptibility gene for this ADR, and this may be due to the fact that the sample size of our study was too small to increase statistical significance of SNPs and genes associated with this ADR.

This study has some limitations. First of all, the main limitation is that our sample size is too small. On the one hand, it could lead to that slight changes in uniformity between samples will significantly affect the results of statistical tests. On the other hand, the Bonferroni correction we used in the statistical analysis is too harsh for the genome-wide association analysis with such a small sample size. Secondly, sinus bradycardia is a complex disease, and for human complex diseases, the pathogenic gene loci may be either very rare among the global population, or the pathogenic effect of most loci is very weak. However, we adopt the microarray, which only includes common variations and few gene loci, to obtain the genotype. Next, usually the disease phenotype is affected by environmental and genetic factors, and environmental factors play an important role in the formation of disease phenotype, especially for complex diseases. The inaccurate identification of the phenotype will reduce the statistical effect of genome-wide association analysis. And the judgment of the clinical phenotype of patients enrolled in this study only considers heart rate changes. Finally, this phenotype may be caused by epigenetic modification and has no relationship with patient’s genotype, but the microarray we used can only obtain the genotypes of the patient in this study.

In conclusion, we performed a preliminary study on 88 Han Chinese SCZ patients with or without sinus bradycardia after taking SGAs in order to identify potential susceptible genes of this ADR. The GWAS result showed that no SNPs reached genome-wide significant association with this ADR. Further, we performed functional analysis, including GO enrichment, gene prioritization and PPI analysis, of top 500 SNP mapped genes. *CTNNA3* showed relatively strong correlation with sinus bradycardia, and this correlation might shed light on the association study of sinus bradycardia induced by SGAs. Future investigations with more samples and high-throughput methods or techniques for epigenetic analysis to obtain more genetic information are warranted to find the significant SNPs or genes or epigenetic modification to predict occurrence of this ADR in the Han Chinese SCZ patients.

## Supporting information

S1 Table List of top 500 SNPs ranked by *P* value from results of GWAS analysis. (XLSX)

S2 Table List of top 500 SNPs mapped genes. (XLSX)

S3 Table List of genes included in PPI analysis. (XLSX)

S4 Table Results of gene ontology enrichment analysis. (XLSX)

S5 Table Full results of ToppGene gene prioritization. (XLSX)

S6 Table Results of gene prioritization of training genes. (XLSX)
